# IHM-DB: a curated collection of metagenomics data from the Indian Himalayan Region, and automated pipeline for 16S rRNA amplicon-based analysis (AutoQii2)

**DOI:** 10.1093/database/baad039

**Published:** 2023-06-03

**Authors:** Abhishek Khatri, Aman Thakur, Ayush Lepcha, Vishal Acharya, Rakshak Kumar

**Affiliations:** Department of Biotechnology, CSIR-Institute of Himalayan Bioresource Technology, Palampur, Himachal Pradesh 176061, India; Department of Biotechnology, CSIR-Institute of Himalayan Bioresource Technology, Palampur, Himachal Pradesh 176061, India; Academy of Scientific and Innovative Research (AcSIR), Ghaziabad 201002, India; Department of Biotechnology, CSIR-Institute of Himalayan Bioresource Technology, Palampur, Himachal Pradesh 176061, India; Academy of Scientific and Innovative Research (AcSIR), Ghaziabad 201002, India; Department of Biotechnology, CSIR-Institute of Himalayan Bioresource Technology, Palampur, Himachal Pradesh 176061, India; Academy of Scientific and Innovative Research (AcSIR), Ghaziabad 201002, India; Department of Biotechnology, CSIR-Institute of Himalayan Bioresource Technology, Palampur, Himachal Pradesh 176061, India; Academy of Scientific and Innovative Research (AcSIR), Ghaziabad 201002, India

## Abstract

Indian Himalayan metagenome database (IHM-DB) is a web-based database consisting of information on metagenomic datasets from various databases and publications that are specifically reported from the Indian Himalayan Region (IHR). The online interface allows users to view or download the dataset-specific information for the respective states, category-wise, or according to the hypervariable region. The IHM-DB also provides an opportunity for the users to access the metagenomic publications from the IHR as well as upload their microbiome information to the website. Additionally, an open-source 16S rRNA amplicon-based automated bioinformatics pipeline, AutoQii2, allows users to analyze the single-end and paired-end raw reads. AutoQii2 provides an automated approach for performing analysis such as quality check, adapter and chimera removal and exploits the latest ribosomal database project classifier for taxonomic assignments. The source code of the AutoQii2 pipeline is available at https://gitlab.com/khatriabhi2319/autoqii2.

**Database URL** https://ham.ihbt.res.in/ihmdb and https://fgcsl.ihbt.res.in/ihmdb

## Introduction

The Indian Himalayan Region (IHR) is stretched across 13 major states of India (about 16.2% of the country’s total geographical area), comprising unique ecological habitats as well as distinct human settlements and their cultural ethics which imparts significant importance to their research and sustainable utilization (1, 2). The IHR is home to a flourished microbiome, which is being explored using metagenomic analysis of diverse habitats such as lakes, geothermal hot springs, glacier ecosystems, caves, compost, the gut microbiome of endemic fauna, the rhizosphere microbiome of endemic flora, as well as the food microbiome of traditional and ethnic culinary of native residents of Indian Himalaya (3–10). The diverse metagenomic studies have revealed the potential microbial resources with range of applications from the production of industrially relevant enzymes to deciphering the ecological role of the microbial community under extreme conditions (3–10). Recent studies on the effect of climate change have apprised the threats of glacier retreat at an alarming rate in the IHR (11). These retreats have resulted in altered hydrological cycles, an increase in carbon dioxide levels and vegetation losses. It has also posed a serious threat to the microbiota native to the glacier ecosystem (11–14). Given the pristine ecological and topological importance of the Himalayas, it is the need of the hour for the estimation, proper documentation and cataloging of the diversity harbored by the IHR.

### The need and value of an Indian Himalayan metagenome database

Among the 36 biodiversity hotspots in the world, the Himalaya has attracted a lot of attention for their unique and rich biodiversity (15, 16). The biodiversity of the IHR has inspired the creation of various databases like ‘Database of vascular plants of Himalayas’ (17), ‘Phytochemical: platform to explore phytochemicals of medicinal plants’ (18) and ‘TeaMiD: a comprehensive database of simple sequence repeat markers of tea’ (19). Even the microbial resources have led to the creation of a database called the ‘North-East India Microbial database’ (20). However, the database specific to metagenomic resources of the IHR is not available, which provides us with the scope for the development of the Indian Himalayan metagenome database (IHM-DB). With the advancements in next-generation sequencing techniques, many metagenomic data have been generated concerning the IHR, but there is no appropriate cataloging and referencing of the generated data. The publicly available databases such as National Center for Biotechnology Information (NCBI), European Molecular Biology Laboratory (EMBL) and Metagenomic Rapid Annotations using Subsystems Technology (MG-RAST) store metagenomic sequences that are more complex and, most importantly, do not categorically provide information. The IHM-DB hosted at https://ham.ihbt.res.in/ihmdb/ and https://fgcsl.ihbt.res.in/ihmdb focuses on the following objectives: (i) gathering all the scattered information about metagenomic data from various databases (NCBI, EMBL and MG-RAST) into a single platform, (ii) segregation of available dataset in a user-friendly manner for easy access and processing, and (iii) providing research literature of metagenomic studies in the IHR. Therefore, the IHM-DB would be the best platform for easy access to all the metagenomic studies carried out in the IHR.

### The IHM-DB

**Figure 1.**
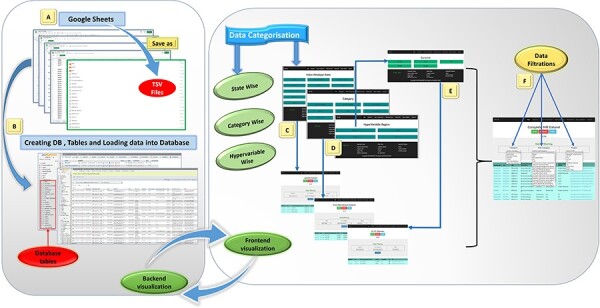
Database preparation, visualization, and workflow from back-end to front-end development of the IHM-DB. (A) Collection of the dataset from publicly available databases. The dataset was manually collected from publicly available databases and stored in Google sheets (tsv files). (B) Creation of database tables in MySQL and loading of collected tsv files. (C–E) Web page visualization of metagenomic data categorized into state-wise, category-wise, and hypervariable-wise classifications. (F) Completion of the IHR dataset on a single page. The users can access the complete metagenome data as well as can use the option of filtration for desirable information.

The IHM-DB is a valuable repository for interested researchers studying the microorganisms within the IHR. It has a comprehensive collection of all the metagenomic datasets and is categorized according to Indian states, category-wise (food microbiome, gut microbiome, hot spring, cave, environmental, and glacier) and the type of variable region (shotgun and amplicon-based sequence) (Figure 1C–E). It is a user-friendly database with easy-to-search, retrieve and submit metagenomics datasets of the IHR. The database will be constantly updated, keeping regular updates on current literature related to metagenomic studies carried out in the IHR. This is the first public release of the IHM-DB, fully functional and expandable, dedicated to metagenomics studies in the IHR.

### AutoQii2 pipeline

**Figure 2.**
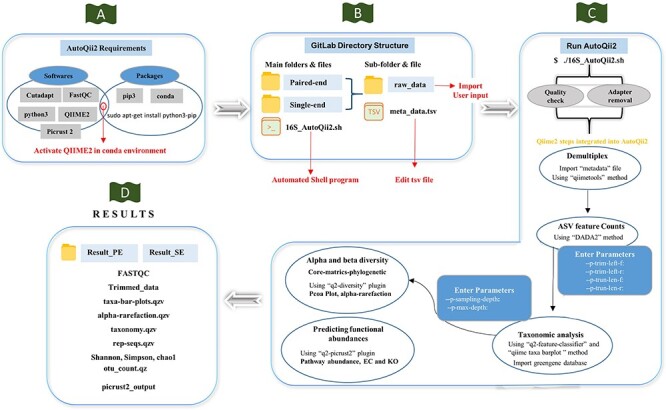
Workflow of AutoQii2 pipeline. (A) Third-party software requirement for AutoQii2. The users are required to install FastQC, cutadapt and QIIME 2 and activate QIIME 2 under conda environment. (B) Open-source code available in GitLab for paired-end (PE) and single-end (SE) analysis. The users are required to edit metadata.tsv and subdirectory ‘raw_data’ available inside the PE and SE folder. ‘16SAutoQii2.sh’ is an automated shell script program. (C) Analysis steps of autoqii2. (D) Results of AutoQii2 Pipeline.

A user-friendly computational automated workflow AutoQii2 was developed for analyzing 16S rRNA amplicon-based datasets (Figure 2). AutoQii2 is primarily designed for eliminating multistep analysis involved in analyzing single-end (SE) or paired-end (PE) reads using Quantitative Insights into Microbial Ecology (QIIME) 2 (21). The command-line interface of QIIME 2 requires a significant number of commands for data processing and analysis. Moreover, researchers are required to investigate the dataset several times for determining the best parameters. This process requires significant effort and generates multiple output files, making the whole process tedious and difficult for interpretation. AutoQii2 is a metabarcoding pipeline that uses an automated interface where interested researchers are required only to enter needful parameters for repeated analysis (Figure 2). AutoQii2 uses FastQC (22), cutadapt (23), and QIIME 2 platforms for performing the quality check, adapter trimming using qiime2-dada2 module, generating amplicon sequence variants (ASVs), taxonomic assignments and functional abundance using q2-picrust2 plug-in. Moreover, the users can access all the output results in a dedicated <results> folder for their convenience. In addition, AutoQii2 provides the users with an interface where the QIIME 2 view result files can be automatically viewed in the browser without the use of a command. This developed workflow provides a fully automated and better data processing of bulk datasets (Figure 2).

## Development of the IHM-DB

### Data collection

**Figure 3.**
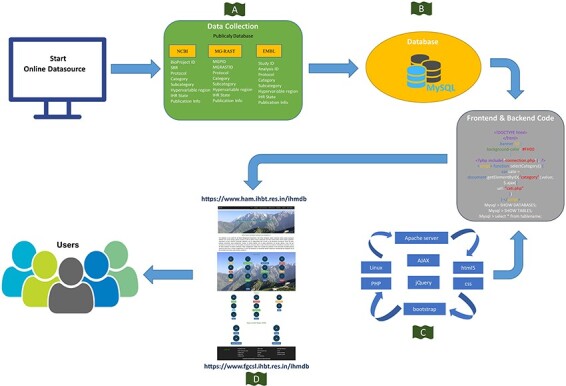
Schematic representation of the IHM-DB. (A) Data collection from publicly available database (NCBI, MG-RAST and EMBL). (B) Storage of all the collected datasets information’s into MySQL database. (C) Languages used for developing the IHM-DB. (D) User-friendly interface of the IHM-DB.

**Figure 4.**
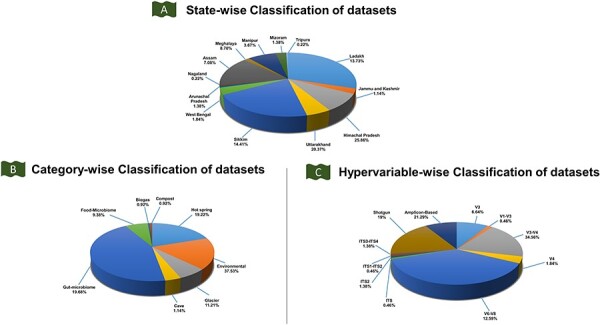
Classification of the IHM-DB. (A) State-wise classification of datasets defines the percentage share of datasets for 13 IHR states. (B) The percentage share for category-wise datasets. The datasets are divided into six categories: food microbiome, gut microbiome, hot spring, cave, environmental, and glacier. (C) The percentage share for hypervariable-wise data. The hypervariable dataset is classified into 11 categories based on shotgun and amplicon-based sequences.

In this study, the metagenomic data were collected from publicly available databases of NCBI (https://www.ncbi.nlm.nih.gov/), MG-RAST (https://www.mg-rast.org) and EMBL-EBI (https://www.ebi.ac.uk/), respectively (Figure 3A). The collected metagenomic datasets (BioProject ID, MGPID, and Study ID) were categorized according to IHR states, categories (glacier, cave, food, hot springs, etc.), and hypervariable regions (shotgun and amplicon-based sequencing) (Supplementary Table S1–S3). In total, 58.67%, 40.67% and 0.67% of metagenomic study data and 80.89% (SRR ID), 18.06% (MG-RAST ID) and 1.05% (ANALYSIS ID) of sample data were collected from NCBI, MG-RAST, and EMBL databases, respectively (Supplementary Table S1; Figure 4). The information regarding published metagenomic research articles was also gathered from the IHR and can be accessed through the ‘publication’ tab in the homepage of the IHM-DB. Additionally, the digital object identifier of the publications pertaining to the collected metagenomic datasets has also been provided in the database.

### Web implementation

**Figure 5.**
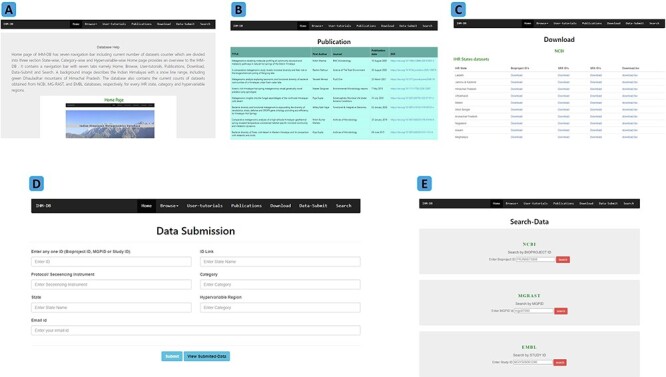
Explanation of navigation tabs in the IHM-DB. (A) Tutorial page with example usage of the IHM-DB. (B) List of metagenomic publications from the IHR. (C) Download page for NCBI, EMBL, and, MG-RAST databases datasets of the IHR data. (D) Data-submit page. The users can submit their data through the Data-Submit page by submitting inputs like NCBI BioProject ID, MG-RAST MGPID, EMBL study ID), ID Link, Protocol/ Sequencing Instrument, Category, State, Hypervariable region, and user email ID. (E) Search page. The users can directly search the dataset by entering BioProject ID, MGPID, and Study ID of NCBI, MG-RAST, and EMBL databases, respectively.

The datasets were first maintained in Google sheets and were converted into tab-separated value (TSV) files (Figure 1A). All the relevant data and information were imported to MySQL (v5.7) database tables (Figures 1B and 3C). The IHM-DB was developed using PHP (v8.1), MySQL (v5.5), and APACHE (v2.4) web server on Ubuntu Linux OS (v18.04). The Apache server communicates with the MySQL relational database to get all the needed information about metagenomics data for the IHR. The front-end database was designed using HTML5, CSS3, Bootstrap, and Java scripts (Figure 3C). The search facilities with keywords like BioProject ID, MGPID, and Study ID were also incorporated into the database (Figure 5E). Each search entry is retrievable from MySQL tables using PHP by the ‘GET’ method, which displayed the user’s search data. The asynchronous JavaScript and XML and PHP server-side scripting were implemented for data filtering in the database.

### Tools included on the IHM-DB

**Figure 6.**
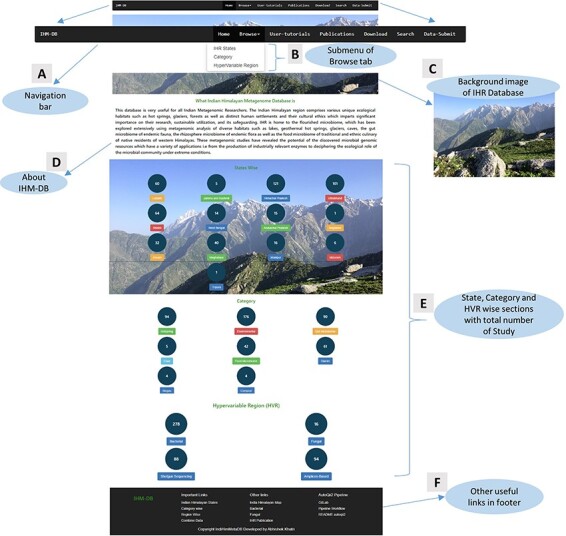
Home page details of the IHM-DB. (A) The home page view of the IHM-DB navigational bar with seven different tabs. (B). Browse tab. It consists of three subtab categories. (C) Background image which describes the Indian Himalayas with a snow line range, including the green Dhauladhar Mountain of Himachal Pradesh. (D) Short description about the IHM-DB. (E) Total number of studies from NCBI, EMBL, and MG-RAST with state-, category- and hypervariable-wise data. (F) Database footer includes other useful links for quick access.

The home page includes the following tools:

**Home**: This online resource homepage provides an overview of the IHM-DB (Figure 6). It contains a navigation bar with seven tabs with browse options (Figure 6A and B). A background image describes the Indian Himalayas with a snow line range, including the green Dhauladhar mountains of Himachal Pradesh (Figure 6C and D). The database also contains the current counts of study datasets obtained from NCBI, MG-RAST and EMBL databases, respectively, for every IHR state, category and hypervariable region (Figure 6E).

**Browse**: This section is divided into three parts with a drop-down tab (Figure 6B) providing the metagenomic information of IHR state-wise, category-wise, and hypervariable-wise as previously mentioned in Figure 1C–E.

**User tutorials:** This section provides a detailed user-guide explaining how to use and browse the database (Figure 5A).

**Publications**: This section provides a list of research publications related to metagenomic studies conducted in the IHR states (Figure 5B).

**Download:** This section provides the download options for the IDs. The users can download a simple list of the IDs in a text or tsv files (Figure 5C).

**Data-Submit:** The users can submit the metagenomic information directly to the IHM-DB through the link (https://ham.ihbt.res.in/ihmdb/data_submission.php) (Figure 5D).

**Search:** A search feature has been included to retrieve specific dataset information from the IHM-DB. The search page specifies three sections, viz., NCBI, MG-RAST and EMBL, which can collect specific data-id information (Figure 5E).

## Development of 16S amplicon-based pipeline integrated with the IHR database

A user-friendly automated bioinformatics pipeline ‘AutoQii2’ for analyzing 16S rRNA amplicon-based datasets has also been developed. This metabarcoding pipeline is helpful for effectively performing automated SE and PE raw datasets analysis. The pipeline has integrated several QIIME 2 steps: (i) metadata preparation, (ii) generating and quantifying amplicon sequence variants (ASVs), (iii) representative sequences alignment, (iv) constructing a phylogenetic tree and (v) alpha and beta diversity test that are essential for analyzing 16S rRNA amplicon-based datasets (Figure 2). Instead of using single QIIME 2 commands in the terminal for a specific task, this pipeline integrated particular commands in a bash script covering significant steps of the QIIME 2 analysis. The users have to directly link the developed shell scripting program that contains 16S rRNA amplicon-based data analysis commands (Figure 2C). The automated pipeline includes steps such as quality check, adapter trimming, generating ASV features, and classification (Figure 2D). The users can access the pipeline through the link (https://gitlab.com/khatriabhi2319/autoqii2) for 16S rRNA amplicon-based data analysis.

### System specifications

AutoQii2 requires a Linux operating system with a minimum of 10 GB space for installation. However, the amount of free disk space and memory can vary depending on the number of samples. The installation directory for AutoQii2 must have sufficient free space to accommodate all input, intermediate, and final data sets, as well as all analysis-specific results.

### Dependencies and software requirements

AutoQii2 depends on different third-party applications (Figure 2A). The users are required to download and install the following software:

1. Python 3 (https://www.python.org)
2. Conda (v 23.3.1) (https://docs.conda.io/en/latest)
3. QIIME 2 (v 2023.2) (https://docs.QIIME 2.org/2021.4/install/native)
4. FastQC (v 0.11.5) (https://www.bioinformatics.babraham.ac.uk/projects/fastqc)
5. Cutadapt (v 1.15) (https://cutadapt.readthedocs.io/en/stable/installation.html)
6. A web browser (Firefox or Chrome) for visualizations of QIIME 2 result outputs.

### Dataset preparation

The users are required to store their raw datasets in SE analysis (raw_data) or PE analysis (raw_data) directories in the FASTQ format (Figure 2B). Next, the details of the raw data, folder path, and other relevant information are required to modify the ‘metadata.tsv’ file (Figure 2B). The users also need to download the greengenes file (gg-13-8-99-515-806-nb-classifier.qza) for taxonomic assignments from QIIME 2 website and place it in the appropriate directory (autoqii2-main folder).

### Workflow

In order to begin 16S amplicon-based analysis using the AutoQii2 pipeline, the users need to install FastQC, cutadapt, and QIIME 2 in the conda environment (Figure 2A). Alternatively, users can download the open source, ‘AutoQii2 pipeline’ repository locally or on a server from the GitLab platform (https://gitlab.com/khatriabhi2319/autoqii2) (Figure 2B). Before beginning the analysis process, there are a few steps that must be followed:

1. Extract the downloaded repository. The ‘16SAutoQii2.sh’ bash script and two subdirectories (PE and SE) will be visible to the users.
2. Store raw data in the “PE/raw_data” directory or SE/raw_data.
3. Edit the ‘metadata.tsv’ file. The users need to provide sample names and folder paths to the raw datasets.
4. Download the greengene database file from QIIME 2 and copy to the repository ‘main-autoqii2’ directory.
5. Execute the script using the command ‘chmod a+x 16AutoQii2.sh’.
6. Run script ‘./16AutoQii2.sh’.

The shell script created as ./16SAutoQii2.sh will ask the users to select the raw data directory (raw_data) and metadata.tsv file through zenity display (dialogue box). This automated script, ./16SAutoQii2.sh, will first perform quality check and adapter trimming from the selected SE or PE raw reads (Figure 2C). This step is required in any next-generation sequencing for basic quality control metrics for raw data and to remove adapter sequences, primers and other types of unwanted sequence from the sequencing reads.

After the completion of a quality check of raw reads, the pipeline will execute the QIIME 2 commands using shell script program and import dataset using the ‘qiimetools’ import plug-in. The dataset will be stored in the compressed ‘demux.qza’ format using qiime2 ‘demux’ plug-in. The AutoQii2 script will open on users’ browser to view number of sequences per sample as well as an interactive quality plot to help decide the truncation length parameters for generating and quantifying ASVs with DADA2 plug-in. AutoQii2 will also perform taxonomic analysis, calculate feature table (includes ASV count data of each sample) and feature data (provides ASV sequences for each sample) using qiime ‘feature-table’ plug-in. The AutoQii2 script will also help in classifying representative sequences using the ‘q2-feature-classifier’ plug-in with greengene reference sequences and provide taxa barplot to visualize the taxonomic profiles of each sample using the ‘qiime taxa barplot’ (Figure 2C). Additionally, AutoQii2 also perform core-metrics-phylogenetic with ‘q2-diversity’ plug-in, which can run a range of alpha and beta diversity program on the datasets (Figure 2C). Further, functional annotation using q2-Picrust2 plug-in will also be carried out for predicting functional abundances in the 16S rRNA amplicon data (Figure 2C). The interface will automatically produce the results that can be accessed from the ‘Results’ directory in the ‘autoqii2-main’ folder (Figure 2D). The details of all the parameters and the method of using it can be found in the GitLab (https://gitlab.com/khatriabhi2319/autoqii2) and GitHub (https://github.com/fgcsl/autoqii2) platforms.

## Conclusions

The IHM-DB is exclusively developed to organize the metagenomics dataset information generated from the IHR. This database has curated the metadata from various resources such as research articles and public databases (NCBI, MG-RAST, and EMBL) and sorted them according to state, category, and hypervariable regions. The end users will have easy access to metagenomic datasets from the IHR sorted into different groups and will also have the option to submit their publically available dataset. Additionally, we have also included an automated user-friendly AutoQii2 pipeline for amplicon-based analysis that is accessible through a database. This database can act as a platform for future expansion and development of a website solely dedicated to the microbiome of Himalaya.

## Supplementary Material

baad039_Supp

## Data Availability

All the data in this manuscript are collected from public databases and can be accessed via https://ham.ihbt.res.in/ihmdb and https://fgcsl.ihbt.res.in/ihmd. The codes and data used for the pipeline are available at https://gitlab.com/khatriabhi2319/autoqii2 and https://github.com/fgcsl/autoqii2.
