# Supplementary material for: IHM-DB: a curated collection of metagenomics data from the Indian Himalayan Region, and automated pipeline for 16S rRNA amplicon-based analysis (AutoQii2)
Source: Database (Oxford). 2023 Jun 3;2023:baad039. doi: 10.1093/database/baad039 (PMC10243898; doi:10.1093/database/baad039)
Supplement: baad039_Supp [file baad039_supp.zip › suppl_data/Supplementary file.docx]

**IHM-DB: a curated collection of metagenomics data from the Indian Himalayan region**

Abhishek Khatri^1^, Aman Thakur^1,2^, Ayush Lepcha^1,2^ Vishal Acharya^1,2^* and Rakshak Kumar^1,2^*****

^1^Department of Biotechnology, CSIR-Institute of Himalayan Bioresource Technology, Post

Box No. 06, Palampur –176061, Himachal Pradesh, India

^2^Academy of Scientific and Innovative Research, Ghaziabad-201002, India.

*Corresponding Authors

**Suplementary Table S1**. Number of studies and samples from NCBI, MGRAST and EMBL of Indian Himalayan region (IHR) states.

| **IHR States** | **NCBI** | | **MG-RAST** | | **EMBL** | | **Total number of study^*^** | **Total number of sample^#^** |
| --- | --- | --- | --- | --- | --- | --- | --- | --- |
|  | Bioproject ID^*^ | SRR  ID^#^ | MGP  ID^*^ | MG-RAST  ID^#^ | STUDY  ID^*^ | ANALYSIS  ID^#^ |  |  |
| Ladakh | 11 | 59 | 1 | 1 | 0 | 0 | 12 | 60 |
| Jammu and Kashmir | 2 | 5 | 0 | 0 | 0 | 0 | 2 | 5 |
| Himachal Pradesh | 15 | 90 | 2 | 26 | 1 | 5 | 18 | 121 |
| Uttrakhand | 10 | 101 | 0 | 0 | 0 | 0 | 10 | 101 |
| Sikkim | 27 | 49 | 15 | 15 | 0 | 0 | 42 | 64 |
| West Bengal | 6 | 12 | 1 | 2 | 0 | 0 | 7 | 14 |
| Arunachal Pradesh | 2 | 15 | 0 | 0 | 0 | 0 | 2 | 15 |
| Nagaland | 1 | 1 | 0 | 0 | 0 | 0 | 1 | 1 |
| Assam | 3 | 5 | 27 | 27 | 0 | 0 | 30 | 32 |
| Meghalaya | 3 | 40 | 0 | 0 | 0 | 0 | 3 | 40 |
| Manipur | 1 | 1 | 15 | 15 | 0 | 0 | 16 | 16 |
| Mizoram | 6 | 6 | 0 | 0 | 0 | 0 | 6 | 6 |
| Tripura | 1 | 1 | 0 | 0 | 0 | 0 | 1 | 1 |
| **Total** | \| **88** \| **385** \| **61** \| **86** \| **1** \| **5** \| **476** \| **150** \| \| --- \| --- \| --- \| --- \| --- \| --- \| --- \| --- \| | **385** | **61** | **86** | **1** | **5** | **150** | **476** |
| * represents number of studies; # represents number of samples | | | | | | | | |

**Supplementary Table S2:** Number of Studies and Samples from NCBI, MGRAST and EMBL of IHR Category wise datasets from IHR.

| **Category** | **NCBI** | | **MGRAST** | | **EMBL** | | **Total number of Study^*^** | **Total number of Sample^#^** |
| --- | --- | --- | --- | --- | --- | --- | --- | --- |
|  | BioProject  ID^*^ | SRR  ID^#^ | MGP  ID^*^ | MGRAST  ID^#^ | STUDY  ID^*^ | ANALYSIS  ID^#^ |  |  |
| Hotspring | 26 | 88 | 1 | 1 | 1 | 5 | 28 | 94 |
| Environmental | 21 | 147 | 4 | 29 | 0 | 0 | 25 | 176 |
| Gut microbiome | 8 | 35 | 55 | 55 | 0 | 0 | 63 | 90 |
| Cave | 5 | 5 | 0 | 0 | 0 | 0 | 5 | 5 |
| Food microbiome | 9 | 41 | 1 | 1 | 0 | 0 | 10 | 42 |
| Glacier | 9 | 61 | 0 | 0 | 0 | 0 | 9 | 61 |
| Biogas | 1 | 4 | 0 | 0 | 0 | 0 | 1 | 4 |
| Compost | 1 | 4 | 0 | 0 | 0 | 0 | 1 | 4 |
| **Total** | **80** | **385** | **61** | **86** | **1** | **5** | **142** | **476** |
| * represents number of studies; # represents number of samples | | | | | | | | |

**Supplementary Table S3:** Number of Studies and Samples from NCBI, MGRAST and EMBL of Hypervariable-region from IHR.

| **Hypervariable-region** | **NCBI** | | **MG-RAST** | | **EMBL** | | **Number of Study^*^** | **Number of sample ids^#^** |
| --- | --- | --- | --- | --- | --- | --- | --- | --- |
|  | Bioproject ID^*^ | SRR ID^#^ | MGP ID^*^ | MGRAST ID^#^ | STUDY ID^*^ | ANALYSIS ID^#^ |  |  |
| V3 | 13 | 43 | 0 | 0 | 0 | 0 | 13 | 43 |
| V1-V3 | 2 | 2 | 0 | 0 | 0 | 0 | 2 | 2 |
| V3-V4 | 27 | 163 | 0 | 0 | 0 | 0 | 27 | 163 |
| V4 | 5 | 15 | 0 | 0 | 0 | 0 | 5 | 15 |
| V6-V8 | 0 | 0 | 55 | 55 | 0 | 0 | 55 | 55 |
| Amplicon-based | 9 | 68 | 3 | 26 | 0 | 5 | 12 | 100 |
| ITS | 1 | 2 | 0 | 0 | 0 | 0 | 1 | 2 |
| ITS2 | 1 | 6 | 0 | 0 | 0 | 0 | 1 | 6 |
| ITS1-ITS2 | 1 | 2 | 0 | 0 | 0 | 0 | 1 | 2 |
| ITS3-ITS4 | 1 | 6 | 0 | 0 | 0 | 0 | 1 | 6 |
| Shotgun Sequencing | 24 | 78 | 3 | 5 | 1 | 5 | 28 | 88 |
| Total | 84 | 385 | 61 | 86 | 1 | 10 | 146 | 482 |
| * represents number of studies; # represents number of samples | | | | | | | | |
